# Dizziness associated with panic disorder and agoraphobia: case report and literature review

**DOI:** 10.1016/S1808-8694(15)30111-7

**Published:** 2015-10-19

**Authors:** João Daniel Caliman e Gurgel, Klinger Vagner Teixeira da Costa, Flavia Nepel Cutini, Krishnamurti Matos de Araújo Sarmento Júnior, Marco André Mezzasalma, Heráclio Villar Ramalho Cavalcanti

**Affiliations:** a MD. Otorhinolaryngology 3rd year resident - SBORL.; b 3rd year resident - Hospital Geral de Bonsucesso.; c Psychologist. Graduate student in Institutional Psychopedagogy.; d M.S in Otorhinolaryngology - UFRJ, MD. Otorhinolaryngologist - Hospital Geral de Bonsucesso.; e M.S. in Psychiatry - UFRJ, Psychiatrist - Instituto de Psiquiatria da Universidade do Brasil.; f MD. Otorhinolaryngologist, Head of the Otorhinolaryngology Service - Hospital Geral de Bonsucesso. Study carried out at the Hospital Geral de Bonsucesso - Rio de Janeiro

**Keywords:** agoraphobia, dizziness, panic disorder

## Abstract

Dizziness is one of the most frequent complaints in both primary and specialized medical care facilities. Many dizzy patients, without a known organic cause, considered as having idiopathic dizziness, may have a psychiatric disorder. Besides, even organic dizziness may cause or exacerbate latent psychiatric alterations. One of the most common disorders associated with dizziness is Panic Disorder with or without Agoraphobia. The aim of this paper is to report a patient’s case and make a literature review on the subject

## INTRODUCTION

Dizziness is one of the most frequent complaints in a medical office, for both primary and specialized care, and continues to be a challenge to clinical thinking. It may be caused by more than two thousand primary or secondary conditions, grouped in more than three hundreds of syndromes.^1^, ^2^ In the United States, dizziness is responsible for over 8 million medical visits per year. Its diagnostic investigation is expensive. According to an American study, it may cost up to US$2,532 to diagnose, and most of the time it is still inconclusive.^3^ Thus, investigators are currently trying to find a controlled and multidisciplinary approach, in order to broadly and directly interfere on the causal and predisposing factors identified.

Under the term dizziness, there are numerous different complaints that go from the symptom of vertigo (characterized by rotational dizziness), all the way to the feeling of unbalance, instability, light headedness or even “near-fainting”.

Many of the patients who complain of dizziness and do not have an apparent cause, therefore considered to have idiopathic dizziness, may have a psychiatric disorder.^4^ Moreover, even dizziness of organic causes may trigger or worsen “latent” psychiatric alterations.^5^

The association between dizziness and psychiatric disorders is already well known, however little studied, mostly because we lack a truly integrated multidisciplinary approach for these patients. As it happens with clinical thinking, the association with psychiatric disorders is treated as a diagnosis of exclusion, that is, cases of dizziness without detectable organic alterations during physical and neurotological exam. These patients end up being considered as carriers of psychiatric-related dizziness, and are referred to the specialist, who takes over the responsibility for treatment, often times without returning to the first physician who saw them.

On the other hand, labyrinthine symptoms in psychiatric patients are often times dealt upon as psychosomatic or neurovegetative manifestations, mostly associated with an anxiety disorder, not requiring specific investigation or treatment.

One of the psychiatric disorders most commonly associated with dizziness is Panic attacks with or without agoraphobia. Somatic symptoms, especially dizziness, are the main traits of these diseases.^6^, ^7^

The goals of this study is to review the literature related to this association, using a case report to illustrate.

## CASE REPORT

K.G.V., female, 23 years old, came to our ward complaining of dizziness that felt like instability, sporadically occurring (approximately 1 spell per week), lasting for a few minutes, especially related to anxiety spells, fatigue, occasional headaches, of one year of onset. The patient denied vertigo, nausea, vomits, ear symptoms or other comorbidities. She reported use of cinarizine 75mg/day without improvement. Once questioned in more details, the patient revealed that her dizziness occurred more frequently in the presence of anxiety spells, peaking within 10 minutes, dyspnea, palpitation, limbs parenthesis and feeling of eminent death. Besides the spells, she was also afraid of leaving home or even staying home alone, fall ill and not have anyone to help her - situations that sometimes caused her dizziness, although of less intensity.

Otoscopy, vestibular and cerebellar tests were all normal. She did not have spontaneous or semi-spontaneous nystagmus.

We then ordered blood tests: CBC, fasting glycemia, urea, creatinine, lipids profile, thyroid hormones, audiometry, impedanciometry and vectoelectronistagmography. Since we raised the issue of a psychiatric disorder (panic disorder associated with agoraphobia), alprazolam was administered in the dose of 0.5 mg/day, and the patient was then referred to a psychiatrist.

On her first return visit, the patient already reported an important improvement in her clinical signs. Complementary tests were all normal. After a suggestion from the psychiatrist, who also corroborated the aforementioned diagnosis, we associated fluoxetine 20mg/day, with total symptoms remission. We then referred the patient to a psychologist.

After 6 months of treatment, the patient remains symptomless and under 10mg/day of fluoxetine (in decreasing doses) and in cognitive-behavioral therapy, being followed by a psychologist in weekly sessions.

## DISCUSSION

Anxiety disorders are the most prevalent psychiatric disorders in the general population and are seen in 15 to 20% of the patients in the general practice ward. Anxiety, in itself, represents a primary psychiatric disorder, it may also be a component of another clinical condition, or secondary to it.^8^

**Chart 1:**
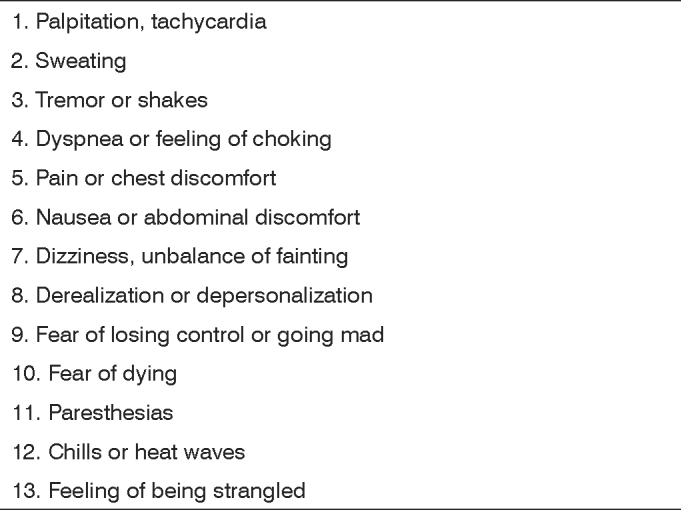
Diagnostic criteria of panic attack.

Source: Diagnostic and Statistical Manual of Mental Disorders, 4th edition.

Panic disorder, the maximum expression of anxiety, is based on the presence of recurrent panic attacks without triggering factors, of sudden onset, usually developing within 10 minutes and unexpectedly resolving within 1 hour. A panic spell is defined as a short period of intense fear and discomfort, during which there is the sudden onset of four or more of the symptoms listed on chart 1.^8^

In some patients, there is anticipated anxiety, resulting from generalized fear and a progressive need to avoid places or situations that could trigger a panic spell. This ends up setting an irrational fear of being in places where the individual may feel like a prisoner or incapable of escaping, thus characterizing agoraphobia.^8^

Many studies have assessed the presence of psychiatric symptoms in patients with vestibular disorders. Just as we saw in our patient, the rates of anxiety and avoidance behavior are considerably high (between 22% and 67%); the rates of vestibular disorders in patients with panic are also high (between 39% and 88%).^9^ Our patient here had normal neurotological exam. Yardley et al.^6^ studied the prevalence of panic disorder symptoms in a sample of patients with dizziness, and how this affects them from the psychosocial stand point. Patients with panic-related dizziness had higher rates of vertigo and agoraphobic behavior when compared to those patients that had only panic or dizziness alone. Moreover, patients with such association and who were employed, half of them left their jobs, representing approximately 10 fold the ratio found among those with dizziness alone and 2 fold when compared to those with panic only. Dizziness duration did not differ among the three groups studied.

Behavioral restriction brought about by a fear of dizziness in patients with vestibular disorders is similar to the phobic avoidance of patients with panic disorder.^10^

In a non-randomized study carried out with 17 patients with “psychogenic dizziness”, 76% had panic disorder and/or agoraphobia, compared to 8% in a sample of 24 patients with severe tinnitus, which is more related to mood disorders such as dysthymia and major depression.^11^ Another study with 75 patients with dizziness revealed that those without vestibular disorder had a significantly higher rate of developing panic spells during their lives.^12^

Jacob et al.^13^ reported that vestibular symptoms during panic spells are not necessarily related to the presence of vestibular dysfunction objectively identifiable; nonetheless, patients with panic disorder associated with agoraphobia have significantly more vestibular disorders than other patients.

Clark^14^ et al. studied a self-help group for dizziness with 103 patients. Of these, 20.4% were diagnosed with panic syndrome, with or without agoraphobia, and 8.7% had only agoraphobia, making up almost 30% with anxiety disorders. Neurotologic tests were not carried out in this study.

Currently, the treatment is carried out by patient education, the use of antidepressive agents, especially the selective inhibitors of serotonin reuptake (fluoxetine, paroxetine, sertraline), benzodiazepines (alprazolam, clonazepam), vestibular rehabilitation and cognitive-behavioral therapy, with excellent results.^5^ With our patient, due to the good response she had with the initial therapy, it was not necessary to submit her to vestibular rehabilitation. We kept only the psychotherapy and the fluoxetine, in weaning doses.

Cognitive-behavioral psychotherapy plays a very important role, especially in keeping the patient free from agoraphobic behavior. In many clinical assays, cognitive therapy proved to be as efficient as drug therapy for light to moderate anxiety disorders. It is based on educating the patient to recognize thought patterns or situations that may trigger, magnify and sustain anxiety, phobic behavior, depression and somatization. Behavioral therapy is done together with cognitive association, through exposing the client, in a hierarchic fashion, to stimuli known to be noxious, aiming at achieving desensitization. Behavioral therapy takes on considerable importance in the treatment of agoraphobia and simple or social phobia.^5^

Because of the large number of differential diagnoses, vestibular complaints are difficult to handle clinically, and therefore require a broad and careful approach. Because of the very prevalence of psychiatric disorders, the otorhinolaryngologist must be ready to identify these symptoms in their patients, screening them for psychiatric follow up. Psychiatric follow up alone is not enough. One should not label the patient as carrier of “psychogenic dizziness”, or conversion disorder, since psychiatric conditions may worsen or even be generated by labyrinthine symptoms. It is necessary to approach the patient in a multi-disciplinary fashion and have the improvement of the patient’s quality of life as the ultimate goal.

Serotonin reuptake selective inhibitors have proven to be efficient in the treatment of panic disorder, with or without concurrent vestibular dysfunction.

Further studies are necessary in order to establish the true relationship between psychiatric disorders and dizziness, such as the prevalence of other psychiatric disorders and dizziness, the role of labyrinthine depressors and serotonin reuptake inhibitors in patients with associated conditions, long term follow up for these patients, amongst others.

## References

[1] Kroenke K, Lucas C, Rosemberg ML, Scherokman B, Herbers JE, Wehrle PA, Boggi JO (1992). Causes of persistent dizziness: A prospective study of 100 patients in primary care. Ann Intern Med.

[2] Ganança FF, Caovilla HH, Ganança MM, Campos CAH, Costa HOO (2002). Tratado de Otorrinolaringologia.

[3] Kroenke K, Mangelsdorff D (1989). Commom symptoms in ambulatory care: Incidence, evaluation, therapy and outcome. Am J Med.

[4] Simon NM, Pollack MH, Tuby KS, Stern TA (1998). Dizziness and panic disorder: a review of the association between vestibular disfunction and anxiety. Ann Clin Psychiatric.

[5] Staab JP (2000). Practical issues in the management of the dizzy and balance disorder patient. Otol Clin North Am.

[6] Yardley L, Owen N, Nazareth I, Luxon L (2001). Panic disorder with agoraphobia associated with dizziness: Characteristic symptoms and psichosocial sequelae. J Nerv Ment Dis.

[7] Katon W (1984). Panic disorder and somatization: Review of 55 cases. Am J Med.

[8] American Psychiatric Association (1994). Diagnostic and Statistical Manual of Mental Disorders.

[9] Yardley L, Luxon L, Lear S (1994). Vestibular and posturographic test results in people with symptoms of panic and agoraphobia. J Audio Med.

[10] Yardley L (1994). Predication of handicap and emotional distress in patients with recurrent vertigo: Symptoms, coping strategies, control beliefs and reciprocal causation. Soc Sci Med.

[11] Simpson RB, Nedzelki JM, Barber HO, Thomas MR (1988). Psychiatric diagnoses in patients with psycogenic dizziness or severe tinnitus. J Otolaryngol.

[12] ullivan M, Clark MR, Katon WJ, Fischl M, Russo J, Dob R (1993). Psyciatric and otologic diagnoses in patients complaining of dizziness. Arch Intern Med.

[13] Jacob RG, Furman JM, Durrant JD, Turner FM (1996). Panic, agoraphobia and vestibular dysfunction. Am J Psych.

[14] Clark DB, Leslie MI, Jacob RG (1992). Balance complaints and panic disorder: a clinical study of panic symptoms in members of a self-help group for balance disorders. J Anxiety Dis.

